# Spatial Ecology of a Resident Avian Predator During the Non-Breeding Period in Managed Habitats of Southeastern Europe

**DOI:** 10.3390/ani14223338

**Published:** 2024-11-20

**Authors:** Draženko Z. Rajković, Daliborka Stanković, Jelena Šeat, Dejan S. Stevanović, Miona V. Andrejević Stošović, Stefan Skorić

**Affiliations:** 1 Department of Biology and Inland Waters Protection, Institute for Multidisciplinary Research, University of Belgrade, Kneza Višeslava 1, 11030 Belgrade, Serbia; daliborka@imsi.bg.ac.rs (D.S.); stefan.skoric@imsi.rs (S.S.); 2 MTA-SZTE ‘Momentum’ Applied Ecology Research Group, University of Szeged, Közép Fasor 52, H-6726 Szeged, Hungary; seatjelena@gmail.com; 3 Faculty of Electronic Engineering, University of Niš, Aleksandra Medvedeva 14, 18000 Niš, Serbia; dejan.stevanovic@elfak.ni.ac.rs (D.S.S.); miona.andrejevic@elfak.ni.ac.rs (M.V.A.S.)

**Keywords:** GPS tracking, habitat choice, home range, kernel utilization, minimum convex polygon, Serbia, *Strix aluco*, tawny owl

## Abstract

Describing home range and resource selection is crucial for understanding ecological needs and creating conservation programs. Still, our knowledge of spatial and behavioural ecology for most species remains limited. Here, we used satellite transmitters to investigate core and home range sizes, habitat selection, and roost characteristics in seven tawny owl males in Western Serbia during the autumn–winter period 2023. Using minimum convex polygon (MCP) and autocorrelated kernel density estimation (AKDE), we found clear variability in core area and home range sizes. Also, adult and heavier males have smaller core area and home ranges than juvenile and lightweight individuals. The Bhattacharyya coefficient showed minor home range overlap in tagged males. The final model for evaluation of habitat selection suggests that the likelihood of owl occurrence was positively correlated with the share of anthropogenic infrastructure and negatively associated with the increase in the proportion of cultivated land within the home range. However, scores of model performance metrics showed moderate predictive accuracy, implying that other unmeasured variables may dictate species presence. Our study illustrates the ecological plasticity and ability of the tawny owl to adapt to a human-modified environment while providing new information about the spatial ecology of this widespread predator in Europe.

## Simple Summary

Using GPS technology, we investigated the spatial ecology of seven male tawny owls in the heterogeneous landscape of Western Serbia during the non-breeding season. Our main goals were to measure home range and core area sizes, understand their habitat choice, and describe roost site characteristics. We caught the owl males and fitted them with lightweight transmitters, which allowed us to track their movements. We found that home ranges varied significantly, with an average size of around 2.2 km^2^. Males with larger body mass exhibited smaller home ranges. Also, we observed minimal overlap in space use between individuals, indicating quite strong territoriality. In terms of habitat selection, increased area of cultivated land substantially reduced the probability of tawny owl presence, while suburban, built-up areas increased it. Our findings provide a better understanding of the home range and habitat selection of tawny owls on the central Balkan Peninsula. This knowledge could have a considerable implication for land-use practices and effective conservation strategies.

## 1. Introduction

All vertebrates need a specific space where they perform essential activities critical for their survival and reproduction [1]. This space typically comprises both the area and habitat they occupy, including the biotic and abiotic components of their distribution range [2,3]. Understanding how animals utilise spatial and temporal resources remains a fundamental goal in spatial and behavioural ecology [4,5]. Knowledge of animal movements and habitat requirements throughout the life span are crucial metrics to understand ecological dynamics and gene flow [6]. These parameters are also critical for developing effective population management and conservation strategies [7,8], especially as most vertebrate species inhabit landscapes significantly altered and shaped by various human activities [9,10].

Animals typically confine their activities to an explicit area described as a home range [11]. The boundaries of this space include the places where they regularly live and gather vital resources for survival, excluding periods of migration, wandering, or unusual movements [11,12,13]. It is important to note that the concept of a home range is distinct from territory, which is a smaller, actively defended space against conspecific or interspecific competitors [11,14]. A correct home range estimate is essential to define habitat use, resource selection, and movement pattern analyses [15,16]. Additionally, estimating home range enables stakeholders and policymakers to formally preserve the critical habitats frequently utilised by species of conservation interest [17,18]. Factors like prey distribution [19], habitat quality [20], competition [21], and physiological needs [22] usually influence the size, shape, and spatial pattern of a home range.

Habitat selection, on the other hand, refers to how animals interact with the various components, conditions, and resources within their environment [23]. Clearly, a particular habitat type may serve for foraging, nesting, roosting, migration, or other life-history traits, reflecting hunting strategies [24], breeding requirements [25], or avoidance of human disturbance [26]. Roosting sites are an integral part of habitat use because they directly influence birds’ spatial and temporal distribution within their home range. Safe and high-quality roosting sites contribute significantly to a bird’s overall fitness and survival [27], affecting their daily and seasonal movement patterns [28]. For example, a secure roosting site can reduce predation risk, allowing birds to spend more time foraging and enhancing their energy balance and reproductive success [15].

Studies regarding home range and habitat selection have a long tradition in ornithological science, e.g., [29,30,31,32]. Over the past few decades, with the significant advancement of sophisticated technologies, the increasing availability of diverse, lightweight tracking devices, and the proliferation of novel statistical techniques and tools, the number of studies on this topic has been increasing rapidly [33,34,35,36]. However, numerous factors, such as easier access to bird individuals (e.g., known nest locations), higher detectability, and research on the relationship between productivity and prey, contribute to more studies of avian home ranges and habitat use during the breeding compared to the non-breeding period. For instance, by May 2024, around 68,600 articles on breeding and 17,900 on non-breeding birds related to home range and habitat use were listed in the Google Scholar repository (https://scholar.google.com/). Furthermore, the majority of avian ecology research is conducted in economically developed countries, frequently focusing on colonial species, diurnal raptors, or species of high conservation interest, e.g., [37,38,39,40]. These geographical and taxonomic biases are probably driven by better funding, infrastructure, and access to advanced research technologies in developed regions. In contrast, nocturnal birds are relatively understudied, especially those in remote areas and economically less developed parts of the world. As a result, there is a considerable gap in our understanding of the spatial ecology of local populations of crepuscular and nocturnal birds like owls (Strigiformes).

The tawny owl (*Strix aluco*; Strigiformes: Strigidae) is a common avian predator widely distributed across the Palearctic region with 11 distinct subspecies [41,42]. Two among them, *S.a. sylvatica* and *S.a. aluco,* inhabit Europe [42,43]. It is the most common and widespread nocturnal raptor on the European continent [41], with a large, stable population estimated to comprise 632–932 hundred thousand pairs [44]. This primarily nocturnal owl is a non-migratory, sedentary species that predominantly breeds in tree holes, cliffs, and buildings or similar cavities [41,42]. Tawny owls inhabit lowland and hilly areas with a preference for broadleaved and mixed forests; however, local populations successfully adapted to less favourable habitats such as city parks, cemeteries, spacious gardens, orchards and tiny forest patches, as well as alpine coniferous forests and rocky outcrops up to 2800 m [41,42]. Southeastern Europe, particularly the Balkan Peninsula, serves as a regional stronghold for tawny owls, accounting for approximately 12.5% of the continent’s population [44]. In addition, in the biogeographical context, the Balkan Peninsula played a crucial role as a Pleistocene refugium of the species on a European scale [45].

In Europe, the tawny owl is an extensively researched owl species [46]. Most earlier works are devoted to its diet [47], breeding biology [48,49,50,51], distribution and vocal activity [52,53,54,55], and habitat relationship [56,57]. Spatial ecology has also been studied but exclusively in developed regions of Western and Northern Europe [50,58,59,60,61]. This research interest is likely due to its widespread distribution, relatively large population, presence in various habitats, and willingness to occupy artificial nest boxes, e.g., [43,48]. On the other hand, there is still much to learn about the biology and ecology of tawny owls, particularly in remote regions in Eastern and Southeastern Europe. Furthermore, earlier studies were limited to an almost entirely different matrix of habitats and climate conditions that differ significantly from those present in Eastern and Southeastern Europe.

The primary aim of our study is to investigate the spatial ecology of tawny owls (subspecies *S.a. aluco*), in particular, to examine the home range, habitat use, and roosting site characteristics in heterogeneous landscapes of central Serbia during the non-breeding period using highly accurate VHF-GPS transmitters. We hypothesise that home range and habitat use differ among the tagged individuals. We assume that males in fragmented and human-altered lowland landscapes would move more between suitable habitat patches in search for food and other resources compared to males inhabiting hilly, far-from-settlements woodland. In short, we expect that “lowland” males have more extensive home ranges than those in hilly areas. Also, based on earlier references, e.g., [41,42,43], we expect that tagged adults would predominantly exploit woodland habitats and juvenile, less experienced males would tend to move more, i.e., have more extensive home ranges and core areas than adults.

## 2. Materials and Methods

### 2.1. Study Area

**Figure 1:**
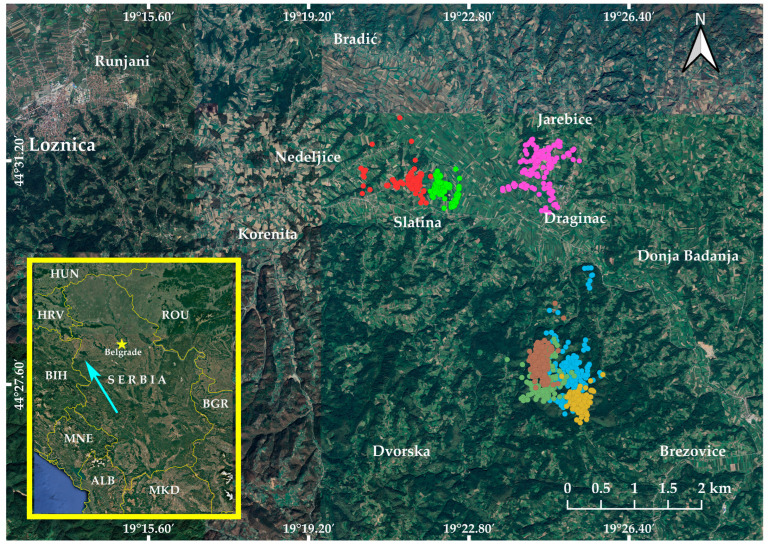
Geographical position and map of the study area, Jadar and Radjevina microregions, Podrinje area, Serbia. The GPS positions unique to each individual male tawny owl are represented by coloured dots. The arrow on the inset map indicates the position of the study area.

Our study took place in part of the Podrinje area (Jadar and Radjevina microregions), covering approximately 950 km^2^ in Western Serbia (Balkan Peninsula, SE Europe). The investigated plot, c. 40 km^2^ in size (approx. centroid N44.4964°, E19.3957°), is located 12 km southeast of Loznica town (Figure 1). The terrain altitude ranges from 120 to 500 m above the sea level, creating a diverse vegetation cover in a mosaic landscape. Overall, the northern section of the study area is a lowland (river valley), while hilly terrain characterises the southern section. In the past, this region was completely covered by deciduous forest. However, centuries of human activities, including logging, land cultivation, and grazing, have significantly altered the forest’s uniformity and age. Nowadays, the northern part of the study area is characterised by typical farmland, with cultivated fields commonly bordered and interspaced with hedgerows, forest patches, small villages along local roads, and several minor rivers and streams. In contrast, the southern section of the study area is predominantly covered by natural and semi-natural broadleaf forests with prevalent species like oak (*Quercus* sp.), linden (*Tilia* sp.), and hornbeam (*Carpinus* sp.) in the lower zones and European beech (*Fagus sylvatica*) in the higher zones, dotted with small watercourses, unpaved roads, and clearings. The human population density in the Podrinje area is about 79 people per km^2^, with an average settlement density of 9 per 100 km^2^ [62]. Although cambisols are the dominant type, the soil composition shifts with altitude change. The climate is temperate continental, characterised by mild summers and cold winters. Meteorological data show an average annual air temperature of 11.7 °C, with precipitation of 858 mm and an average of 2041 h of effective sunlight per year [63].

### 2.2. Capturing, Tagging, and Data Collection

Prior to capturing, we surveyed the study area for tawny owl territories in mid-October 2023. Except for the far north of Europe, this period of a year provides adequate vocal responses from territorial tawny owls, as juvenile owls establish territories and adults defend theirs in preparation for the spring nesting season [52,53,54]. Since the tawny owl’s acoustic activity is notably reduced in windy [55] and rainy weather [64], all field surveys were conducted on calm, dry evenings. The survey methodology followed the call-broadcast techniques demonstrated by [65]. The positions of calling owls were plotted on a Google Maps app (https://play.google.com/store/apps/details?id=com.google.android.apps.maps&pli=1, accessed on 25 August 2024) with accompanying data like type of response and sex.

**Figure 2:**
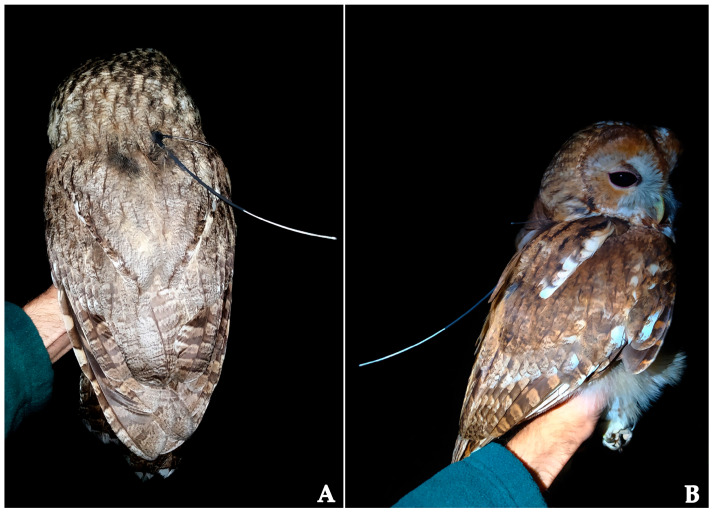
Grey (**A**) and rufous (**B**) colour morphs of male tawny owls equipped with X-attached VHF-GPS transmitters, photographed just before release.

We used the gathered data on the spatial distribution of eleven discovered territories, defended by a pair or a male, to identify suitable microlocations (e.g., small clearings, prominent ridges, or crossroads) for capturing. A vertical ornithological mist net (Ecotone, Gdynia, Poland) with five shelves and a 70 mm mesh size (3.2 × 18 m) was used to capture the owls. To attract the owls to the mist net, we used the fact that tawny owls fiercely defend their territory against conspecific intruders [49,66]. Thus, we used a decoy, a handmade, black-painted Styrofoam owl model placed at the midpoint of the mist net, accompanied by the broadcast of the male’s territorial hooting from a loudspeaker placed underneath. We set the mist net in the evening and checked it every 20–30 min. All seven owls in this study were captured from late October to early November 2023. Before tagging the males, all individuals were aged, sexed, and ringed and morphological traits were measured. Also, the body mass of each individual was recorded (Table 1) using a spring scale with a 1000 g capacity and an accuracy of ±2 g (PESOLA, Chur, Switzerland). Owls were ringed with distinctively coded aluminium butt-end rings (Natural History Museum, Belgrade, Serbia). Age and sex were determined using the moult stage, morphological measurements [67] and (D.Z. Rajković, unpublished data), and voice expressions in the field [58] during the capturing event. Finally, we equipped captured individuals with lightweight PinPoint VHF-GPS transmitters (models 120 and 240; 5.3 and 8 g; Lotek Wireless Inc., Wareham, UK) with a specimen-unique frequency. The transmitters were X-attached to the birds in a thoracic configuration beneath the plumage by stitching and adhering a 6.3 mm wide Teflon ribbon harness to the transmitter [68,69] (Figure 2).

Single transmitter attachment took 21 to 32 min, respectively. Transmitters were cryptically coloured to prevent potential predation [70]. After tagging and a few minutes of habituation, all trapped individuals were released close to their capture locations. 

Although there is no sufficient evidence that proportionally heavier devices possessed more significant consequences on the tagged specimen, e.g., [71], as a rule of thumb, the weight of our transmitter was considerably lower than the typically recommended 3% of the birds’ weight (1–1.6%, respectively) in order to minimise the possible risk of adverse tag effects [72,73]. Furthermore, Sunde [74] reported no negative effects associated with transmitter attachment and tawny owl survival and reproduction parameters.

The VHF-GPS transmitters were pre-programmed to provide timestamps and to record longitude and latitude five times per day: one fix during daylight (midday) and four fixes from dusk till dawn (6:00 p.m., 10:00 p.m., 2:00 a.m., 6:00 a.m.). This schedule was designed to ensure the record of daytime roosting sites and nighttime movements for home range calculations. The data collected and stored by the internal transmitter memory were downloaded remotely using a hand-held 3-element Yagi antenna and a portable PinPoint VHF receiver (Lotek Wireless Inc., Wareham, UK). For further computer analysis, we transformed all collected locations into a rectangular coordinate system-UTM coordinates (WGS 84, EPSG: 32,630).

### 2.3. Estimation of Home Ranges and Spatial Overlap

In the initial step, the collected data were manually sorted and filtered in Microsoft Excel and visually inspected in Google Earth Pro. We filtered collected movement data in order to exclude all GPS fixes of limited accuracy (less than four satellites) [75,76]. Also, we discarded the fixes from the first night because of the possible influence of the freshly mounted transmitters in the case capturing and handling stress interrupted owls’ normal behaviour and activity patterns. We used a probability utilisation distribution (UD) to estimate the home range and core area. We estimated the UD by employing two different techniques: Minimum Convex Polygon (MCP) [77,78] for 95% isopleth and the Autocorrelated Kernel Density Estimation (AKDE) [79] for 95% (hereafter home range) and 50% isopleth (hereafter core area). However, we only report MCP to enable comparisons with previously published data; this geometric estimator is sensitive to outliers and marginal fixes and tends to underestimate home range areas on autocorrelated data [80,81]. Considering the existing flaws of MCP, we perform AKDE to provide additional information on this matter. This technique represents a relatively novel statistical concept used in ecology, primarily describing the likelihood that an animal can be found at a particular location or area over a given time span [82,83]. We preferred AKDE over other frequently used estimators in ecological studies because it mitigates different biases that can heavily affect home range estimates, see [76,84,85,86]. In brief, AKDE improves upon traditional kernel density estimation (KDE) methods by factoring in the time dependency and autocorrelation of the animal movement data. Also, this method reduces the bias and variance in home range estimates that can be significant in traditional KDE when used with autocorrelated data [84,87].

In order to reproduce the home range, core area, and overlap analysis employing the AKDE method, we used R software version 4.3.2 [88] enhanced by a robust framework of continuous-time stochastic movement models, ctmm web app (https://ctmm.shinyapps.io/ctmmweb/) (accessed on 25 August 2024) [89,90]. After importing the dataset, we employed the integrated ‘Filter Outliers’ tool to mitigate the impact of potential data skewness that could skew the analyses. Further, we assessed the autocorrelation structure of a tracking dataset and temporal stability (range residency) of the tawny owls’ home ranges through variogram analysis [86]. Model selection was performed using a maximum likelihood approach, comparing the suitability of several offered stochastic movement models: Brownian Motion (BM), Integrated Ornstein–Uhlenbeck (IOU), and Ornstein–Uhlenbeck Foraging (OUF). The most supported movement model was selected based on the lowest Akaike’s Information Criterion value [91,92]. As the GPS data collection schedule was slightly altered due to habitat-related signal loss, we used the weighted AKDE, in particular, the ‘Optimal Weighting’ tool [79,86]. This choice allowed us to recalibrate the weightings of the data points, thereby correcting for any inconsistencies introduced by the changes in the fixed collection protocol [79]. Lately, we quantitatively defined and visualised the home ranges using the AKDE approach, mapping the 95% and 50% confidence intervals of the home range contours. The movement distance of tawny owl males was calculated using the distHaversine function in the geosphere package [93].

To quantify the extent of spatial overlap among tagged owl males, we applied a robust, pairwise metric, the Bhattacharyya coefficient (BC) [94,95], also in the ctmm interface. The data for each of the seven owl males were organised into a paired matrix to form dyads (N = 21), which were subsequently utilised to determine home range overlap. A dyad refers to a pair of specific individuals, regardless of whether their home ranges showed any spatial overlap [96]. The BC estimates range from 0 (indicating no overlap) to 1 (complete overlap).

### 2.4. Defining Habitat Selection and Roosting Sites

After collecting and processing GPS location fixes, we quantified habitat selection by tawny owl males at the third-order selection as proposed by Johnson [97]. To delineate habitat classes within each home range, we employed a combination of field surveys, Google Earth Pro, and QGIS version 3.14 (http://test.qgis.org/html/en/site/index.html) (accessed on 27 May 2024). For simplicity and ecological relevance, we grouped habitat into three main categories: forest, cultivated land, and suburban areas. Forested and suburban areas with buildings provide shelter, roosting sites, foraging, resting, and perching opportunities. At the same time, open habitats such as cultivated fields together with grasslands support large populations of rodents and other small mammals, the tawny owl’s preferred prey in Europe [41,42,43,47]. In addition to environmental, we also included altitude as a topographic variable.

In the next step, we applied a logistic resource selection function (RSF) [98] using a generalised linear model (GLM) with logistic regression. The RSF compares environmental and other conditions at animal-occupied sites with randomly selected available sites, which are the available resources within the study area. This use-availability method uses a weighted distribution to estimate the likelihood that an animal, in our case owl, will select a specific resource based on environmental characteristics [98]. As the RFS model needs used and available points, we generated available points in QGIS randomly spaced within home range borders. We generated the same number of available points as the used points.

Before GLM modelling, we calculated the Spearman’s correlation matrix to screen for potential high collinearity (r > 0.7) among the four predictor variables. Only forest and cultivated land showed a high correlation (Spearman’s r = −0.86). As a result, we excluded the forest variable from the model-building process due to its weaker bivariate association with the dependent variable (r = 0.15 for forest, r = −0.20 for cultivated land). Also, in order to further quantify the degree of multicollinearity, we checked VIF values, which confirmed no significant multicollinearity problem (VIF = 1.12–1.34). Subsequently, we developed all possible combination models (N = 7) based on single, two, or three retained predictors (cultivated land, suburban, and altitude). To choose the best model, we used a widely applied Akaike information criterion (AIC) [91]. We addressed the model selection uncertainty by Akaike weight, which can be explained as the probability of the model being the best in the set of candidate models [91].

Model accuracy was tested using the AUC statistic [99], which evaluates the model’s ability to distinguish used from available locations. AUC values range from 0.5 (random discrimination) to 1.0 (perfect prediction). In order to avoid overfitting and calculate predictive accuracy, we conducted a ten-fold cross-validation with the DAAG package [100], dividing the dataset into ten random subsets, each used once for testing while the other nine were used to fit the model. Roosting site height (m) was measured directly in the field with a laser distance measurer (model PARKSIDE PLEM 50 C3, Parkside, Neckarsulm, Germany; accuracy 1.5 mm) to the nearest 10 cm.

### 2.5. Additional Statistical Testing

In addition to the statistical procedures described in the previous sections, we employed descriptive statistics (measures of central tendency) and several standard statistical tests to assess correlations and differences and to summarise our findings. Due to their robustness, we used Welch’s *t*-test, Kruskal–Wallis H test and Fisher’s Exact Test with the Haldane–Anscombe correction. In particular, Fisher’s Exact Test was used to examine the association between habitat (hilly vs. lowland) and roost location type (natural vs. anthropogenic). All mentioned tests are useful when dealing with small sample sizes or when some cells in a contingency table have low or zero counts, as in our study. Mean values are presented with their corresponding standard deviations for all numerical data. Unless otherwise specified, the cut-off point implying statistical significance was *p* < 0.05. All performed statistical tests were two-tailed and were conducted using R [88].

## 3. Results

### 3.1. Home Range and Core Area Estimates

The present study tracked seven GPS-tagged tawny owl males between 35 and 58 days (mean = 47; SD = 3.4). Altogether, 1440 refined, effective locations were obtained (mean = 205.7; SD = 19.6; range = 144–262). The tracking duration (in days) did not show a significant correlation with the size of the core area (log-transformed; Spearman’s correlation r = −0.18, df = 5, *p* = 0.7) or home range (Spearman’s correlation r = 0, df = 5, *p* = 1). Also, there was no correlation between the number of GPS fixes per owl male and the estimated home range size (Spearman’s correlation r = −0.11, df = 5, *p* = 0.82).

**Figure 3:**
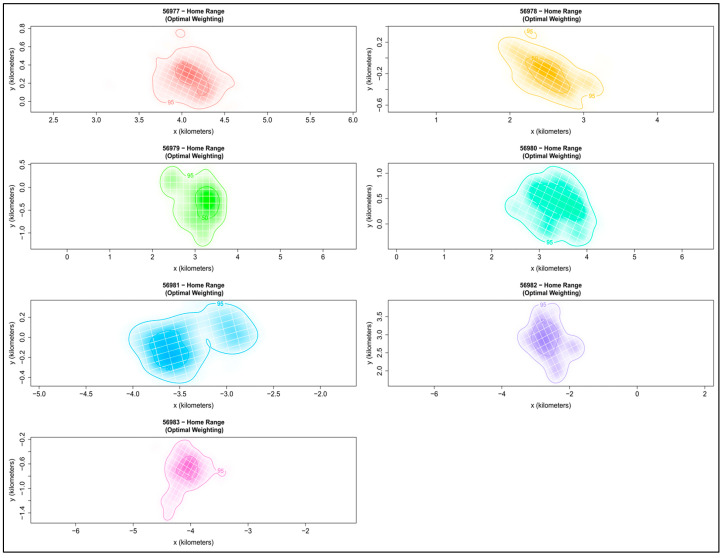
Non-breeding home range estimates for seven tawny owl males, *Strix aluco*, tracked in Jadar and Radjevina (Podrinje area, Western Serbia) during the period of autumn–winter of 2023. The outer boundary represents the 95% AKDE, while the inner polygon delineates the 50% AKDE core area.

The home ranges of the tracked males varied significantly in terms of size and shape (Table 2, Figure 3). The mean home range per male individual was 157.04 ha (SD = 94.69; range = 38.6–285.65) for 95% MCP isopleths. Moreover, the mean size of the home range (95% AKDE) was 217.78 ha (SD = 145.95; range = 56.36–462.23), which is equivalent to approximately a 0.8 km radius around the core area centroid. Range sizes for core area (AKDE) were from 9.95 to 89.74 ha with a mean of 40.43 ha (SD = 31.74; Table 2).

A solid and highly significant relationship exists between core area and home range size (Spearman’s correlation r = 0.96, df = 5, *p* < 0.001). Specifically, a larger core area is strongly associated with a more extensive home range. Additionally, there is a strong negative relationship between individual body mass with home range (Spearman’s correlation r = −1, df = 5, *p* < 0.001) and core area size (Spearman’s correlation r = −0.96, df = 5, *p* = 0.001).

The results of the *t*-test fail to reject the null hypothesis and indicate an absence of a significant difference between lowland and hilly males in home range size (t = −0.06, df = 4.82, *p* = 0.95, 95%CI = [−342.10, 326.23]) or core area size (t = −0.06, df = 3.84, *p* = 0.95, 95%CI = [−85.64, 81.87]). The testing was supported by minor and non-significant effect sizes for both the core area and home range (Hedges’ g = −0.04), suggesting negligible similarity in mean values between the two relief classes.

**Figure 4:**
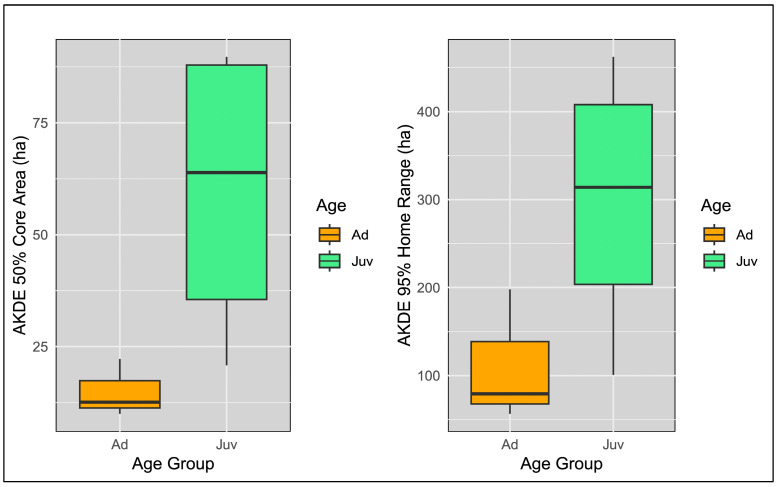
Comparison of core area (50% AKDE) and home range (95% AKDE) estimates between adult (Ad) and first-year (Juv) tawny owl males, *Strix aluco*, during the non-breeding season in Jadar and Radjevina microregions, Podrinje area, Western Serbia.

Further analysis also revealed no significant difference in means between juvenile and adult individuals for home range (t = 2.03, df = 4.46, *p* = 0.11, 95%CI = [−58.12, 431.32]) and core area (t = 2.54, df = 3.28, *p* = 0.08, 95%CI = [−8.76, 98.06]). Despite the lack of statistical significance, relatively high values of effect size (Hedges’ g = 1.18 and g = 1.4) and box plot inspection suggest differences in age classes and the superiority of juvenile individuals in home range and core area sizes (Figure 4).

Based on the collected timestamps from transmitters, we found that the average Euclidean movement distance per night for a single male owl was 1088.7 m (SD = 618; median = 1034; range = 45–3082). A strong, positive correlation was detected between the mean night travel distance per individual and the size of the home range (Spearman’s correlation r = 0.82, df = 5, *p* = 0.02). However, there was no significant difference in the travelled distance between owl males in lowland and hilly areas (t = 1.04, df = 298, *p* = 0.3, 95% CI = [−0.04, 0.12]). On the other hand, on average, juveniles tended to move more during a single night compared to adults, and this difference was highly significant (t = −5.99, df = 238.3, *p* < 0.0001, 95% CI = [−0.29, −0.15]). Across the four buffer zones (0–250, 251–500, 501–1000, >1000 m), nearly 60% of movements were within a 250 m radius measured from the core area centre. The results of the Kruskal–Wallis H test showed a statistically significant difference in median distance movement from the core area centre across the tagged males (χ^2^ = 293.44, df = 6, *p* < 0.0001).

The analysis of the BC overlap index revealed varying degrees of spatial overlap between the home ranges, with overall minimal intersection observed between neighbouring males. The BC values ranged from 0 to 0.45, with the strongest overlap noticed between males coded 56978 and 56979 (Table 3). In general, juvenile males and those which inhabit hilly areas tend to have more spatial overlap than adult individuals and those from the lowland part of the study area. However, the average overlap BC index was just 0.06 (median = 0; SD = 0.15), representing a minor level of home range sharing among the studied individuals (Table 3).

### 3.2. Habitat Selection

According to GLM modelling, Model number 4, which included the variables Cultivated land and Suburban, offered the most proper balance between model complexity and goodness-of-fit. Therefore, it was selected as the best-performing model (Table 4 and Table 5).

Nonetheless, the model rankings based on AIC values offer some information about the relative significance of predictors. For instance, Model 7, which ranked second, suggests that topography, combined with other habitat features, may provide valuable information about the potential presence of tawny owl individuals. Notably, cultivated land represents a consistent predictor variable among the generated models with the lowest AIC and Δ*_i_*AIC values.

As expected, the likelihood of tawny owls choosing a particular habitat during their nocturnal activities was strongly linked to the amount of agricultural land in the area, with less cultivated land inside the home range being preferred. Despite this, the AUC score (0.601) for the best model indicated only moderate predictive accuracy. Additionally, McFadden’s pseudo-R-squared of 0.043 indicates that the model explains only a tiny portion of the data variance, implying that other unmeasured predictors may significantly influence species presence within the home range boundaries.

### 3.3. Roosts

During our research, we discovered 15 different roosting locations used by tagged tawny owls. Three individuals consistently used the same roost during the entire study period. The remaining four males moved between roosting sites 26–297 m apart (mean = 174.4; median = 200; SD = 93.8). On average, each male had 2.1 roosting spots (median = 2; SD = 1.2; range = 1–4). Seven roosting sites were situated in holes in tree trunks (46.6%), four inside buildings (26.7%) and four were positioned outside (26.7%) on branches close to the main trunk. With a single exception, all roosts were situated within the core area boundaries. The distances between the nearest neighbouring roosts were closer in males inhabiting higher altitudes (mean = 485; median = 485; SD = 102.8; range = 396–574 m) compared to neighbouring roosts of lowland males (mean = 1399.7; median = 552; SD = 1468.2; range = 552–3095 m).

The mean height of the roosting places from the ground was 10.5 m (median = 10; SD = 3.9; range = 2–17). In the lowlands, the mean height of the roosting place was 7.6 m (median = 8.5; SD = 2.7; range = 2–10), while in a hilly landscape, it was 13.7 m (median = 14; SD = 2; range = 11–17). Statistically, tracked males in the lowlands tend to roost at lower heights compared to those in the hilly landscape (t = 5.06, df = 12.7, *p* < 0.001, 95%CI = [3.48, 8.69]; Hedges’ g = 2.41). Conversely, there was no significant difference in roosting height between the two age classes (t = 1.43, df = 8.56, *p* = 0.190, 95%CI = [−1.73, 7.47]). The effect size was moderate (Hedges’ g = 0.73), suggesting a modest difference between the sample means.

The analysis of Fisher’s Exact test yielded a *p*-value of 0.080, showing that the association between these variables was insignificant. The odds ratio was estimated as infinite, with a 95% confidence interval ranging from 0.810 to infinity, which probably reflects the complete absence of anthropogenic origin roost sites in hilly areas. In sum, although the results approached very close to statistical significance, the data do not provide sufficiently strong evidence of an association between analysed variables.

## 4. Discussion

Here, we present the results of the first study on the home range and habitat selection of tawny owls in the region of Southeastern Europe. We found considerable variation in home range and core area sizes among tagged males of tawny owl in the mosaic landscape of Western Serbia during the non-breeding period. This variability suggests that the owls, on an individual level, exhibit a high level of adaptability and flexibility in habitat utilisation. Also, we did not notice statistical significance in the size of the core area and home range between individuals inhabiting lowland or hilly regions, which indicates that certain topographical variability within the study area does not influence spatial use patterns. A possible explanation is that both lowland and hilly environments in our study area offer comparable resources (e.g., prey availability). Even though there is no statistically significant difference in home range size between juvenile and adult individuals, box plots of AKDE values show a biologically meaningful discrepancy where juveniles tend to have a more extensive home range than adults (see Figure 4). This inconsistency between the visual representation and statistical results is likely due to the small sample size [101], which reduces the statistical power for recognising a natural variation in home range sizes.

This assumption is also based on the more extensive movement of juvenile males during the average night compared to adults. Such conviction aligns with the hypothesis that juvenile owls may require larger ranges to secure sufficient resources and energetic needs, as they are still establishing their territories and developing foraging skills [102,103]. In addition, such behaviour might be a fundamental mechanism for reducing competition with adults, which, in the end, could influence population dynamics.

Previous works on avian predators demonstrated a significant link between home range size and extrinsic factors like habitat quality and landscape heterogeneity [7,19,20,104,105]. In our study, home range size may be associated with habitat quality, as avian predators in poorer-quality habitats may need to travel further to meet their needs, and vice versa [59,102]. From a predator’s perspective, key components of habitat quality include prey density, abundance, availability, spatial distribution, suitable resting sites, low predator pressure, social structure, territoriality, or a combination of these components [15]. Given that our study area provides a sufficient number of roosting places and that significant predators are completely absent (apart from the Northern Goshawk *Accipiter gentilis*), the factors that presumably contribute to the variability in home range size are prey availability and territoriality. Indeed, owls’ home ranges barely overlap, particularly among adults, suggesting assertive territoriality behaviour in our study area. Thus, territory guarding and defence could partially explain home range patterns. This is consistent with earlier studies of tawny owls, which have proved a high level of territoriality and minimal home range overlap as part of a strategy to reduce competition [58,59,60]. Interestingly, juveniles and individuals in hilly areas showed slightly more spatial overlap than their adult and lowland counterparts, indicating that territorial boundaries may be more fluid among young, less experienced individuals or in more rugged terrains.

Despite variability and different home range estimation methods, our home range sizes are in accordance with those reported in earlier studies, where ranges vary from 16 to 545 ha [50,59,60,106,107]. Also, a positive correlation between core area and home range size reaffirms the hypothesis that the size of the core area is a pretty reliable predictor of overall home range proportions in avian predators [59,94]. Additionally, it may also reflect the influence of habitat suitability and heterogeneity inside home range boundaries, as these factors can significantly shape the spatial use patterns. Based on the present study and considering that home range size stays relatively consistent throughout the year [60,61], we can conclude that in central Balkans, home ranges are likely to range between 150 and 220 hectares, regardless of habitat quality. This information may be essential for refining regional population size estimates, improving the interpretation of nocturnal survey results and supporting better habitat management and conservation planning.

The negative bivariate relationship between body mass and home range size is particularly noteworthy. This finding could reflect compensatory behaviour, where individuals with lower body mass may need to cover more ground to meet their energy requirements, a pattern already documented in tawny owls [58,108]. Simply, larger home ranges result in greater energy expenditure during foraging and movement. This increased effort could then lead to reduced body mass, especially if prey or other critical resources are limited.

Based upon current knowledge, the tawny owl prefers various types of forest habitats including small patches surrounded by open areas [41,42,43,49,58]. Within forest ecosystems, it primarily favours deciduous and mixed woodlands at lower altitudes [41,42,43,109]. Also, a few studies have shown that altitude and topography are significant determinants of distribution and habitat usage in regions with complex topography, e.g., [109]. However, in the present study, we achieved slightly different results. First, it seems that extensively forested areas did not play such a significant role in meeting the habitat demands of the central Balkan population of tawny owls, at least during the non-breeding period. Second, as for home range size, we found that altitude is not a reliable predictor of owl presence in the studied area. Third, and likely most importantly, we discovered that cultivated land and suburban areas appeared to be the most influential predictors at the third order of selection (Table 5). Altogether, our results suggest that cultivated land surfaces strongly decrease while suburban infrastructure modestly increases the likelihood of tawny owl presence in mosaic landscapes of the central Balkan Peninsula. This may be a consequence of recent agricultural intensification in the region [110], which has led to the removal of isolated trees and a reduction in the surface of hedgerows and treeline rows. Also, these findings support the idea that other habitats and their fragments can be important factors for tawny owl survival, compensating for the absence of extensive forest cover. Several previous studies have also highlighted the ecological plasticity of tawny owls in adapting to human-modified environments and built-up areas [43,51,56]. Consequently, this could reflect regional differences in habitat structure and resource distribution, where small patches, fragmented habitats, and edge effect offset the reduction in primary habitat cover, i.e., woodlands, as concluded in a few other studies [57,108]. However, the interpretation of the results on habitat selection in the central Balkans needs to be further examined with a larger sample size, considering the relatively low explanatory power of our final model. In addition, future studies should incorporate additional predictor variables, such as prey availability and abundance, predation pressure, competition with other nocturnal rodent predators, and human disturbance, into the modelling process.

Daytime roosting sites are an integral part of habitat selection, as location choice, position, and features could significantly impact survival and overall fitness. For example, in ideal conditions, these sites help diminish owls’ exposure to harsh weather conditions and potential predators [111,112,113]. In Western Serbia, tawny owls showed a clear preference for well-sheltered microlocations. Most roosts were found in tree cavities or inside buildings, clustered in core areas. The significantly greater roosting height detected in hilly areas compared to lowland likely reflects the structural characteristics of the various habitat elements in the study area. We assume that taller and thicker trees in these areas provide safer resting places and a better vantage point of the surroundings. This behaviour may also be influenced by reduced human disturbance or, more likely, by the higher availability of suitable natural sites in hilly landscapes. The height of the roosting site in natural cavities in our study area is in agreement with the findings of a detailed study from Poland but is, on average, significantly higher than in the temperate forests of Ukraine [113]. These regional differences suggest that the diversity, age, and density of tree species dictate owl roosting site choice in natural environments, further accenting their broad phenotypic plasticity regarding habitat requirements.

## 5. Conclusions

Our study represents the first analysis of home range and habitat selection for tawny owls in Southeastern Europe. We found high individual variability in core area and home range size. While forest habitats are traditionally considered essential for tawny owl reproduction and survival, our study demonstrates that other habitat types, such as cultivated lands and suburban areas, can play important roles, at least in certain regions and periods of the year. These findings underline the ecological adaptability of tawny owls as they adjust to various landscapes, especially in areas altered by human activities. Future research should focus on integrating additional predictors, such as prey abundance, habitat structure, or climate and human disturbance, to capture the complexity of habitat selection and assess how these factors interact with forest availability in shaping tawny owl home ranges.

## Figures and Tables

**Table 1:** Summary data of satellite-tracked tawny owls (*Strix aluco*) in the study area. Ad—adult individual (hatched before study year), Juv—juvenile individual (hatched during study year); HFL—hilly forested landscape; LAL—lowland agricultural landscape with scattered forest patches.

Individual ID	Capture Date	Age	Sex	Body Mass (g)	Habitat	Collected Fixes (n)	Tag Model	Transmission Period (Days)
Male_56977	28 October 2023	Ad	Male	488	HFL	269	240	55
Male_56978	28 October 2023	Juv	Male	474	HFL	264	240	54
Male_56979	29 October 2023	Juv	Male	446	HFL	180	240	46
Male_56980	29 October 2023	Juv	Male	417	HFL	281	240	58
Male_56981	30 October 2023	Ad	Male	486	LAL	213	120	45
Male_56982	30 October 2023	Juv	Male	436	LAL	162	120	37
Male_56983	2 November 2023	Ad	Male	467	LAL	157	120	35

**Table 2:** Core area and home range estimates for seven tawny owl, *Strix aluco*, males in the Jadar and Radjevina microregions (Podrinje area, Western Serbia), based on Minimum Convex Polygon (MCP, 95%) and Autocorrelated Kernel Utilisation Distribution (AKDE, 95% and 50%).

Individual ID	MCP (ha)	AKDE Core Area (ha)	AKDE Home Range (ha)
Male_56977	38.60	9.95	56.36
Male_56978	64.33	20.80	100.84
Male_56979	170.50	40.43	238.08
Male_56980	285.65	87.33	462.23
Male_56981	60.65	12.59	79.22
Male_56982	254.52	89.74	389.85
Male_56983	225.05	22.20	197.87

**Table 3:** Home range (95% AKDE) overlaps of male tawny owls *Strix aluco* for the 21 dyads at the 95% confidence level.

Dyad	Individual I	Individual II	Home Range Overlap	95% CI
1	Male_56977	Male_56978	0	0
2	Male_56977	Male_56979	0.02	0.01–0.04
3	Male_56977	Male_56980	0.32	0.27–0.37
4	Male_56977	Male_56981	0	-
5	Male_56977	Male_56982	0	-
6	Male_56977	Male_56983	0	-
7	Male_56978	Male_56979	0.45	0.37–0.53
8	Male_56978	Male_56980	0.11	0.08–0.15
9	Male_56978	Male_56981	0	-
10	Male_56978	Male_56982	0	-
11	Male_56978	Male_56983	0	-
12	Male_56979	Male_56980	0.43	0.34–0.52
13	Male_56979	Male_56981	0	-
14	Male_56979	Male_56982	0	-
15	Male_56979	Male_56983	0	-
16	Male_56980	Male_56981	0	-
17	Male_56980	Male_56982	0	-
18	Male_56980	Male_56983	0	-
19	Male_56981	Male_56982	0	-
20	Male_56981	Male_56983	0.03	0.02–0.04
21	Male_56982	Male_56983	0	-

**Table 4:** The Akaike information criterion (AIC) and performance metrics for tawny owls’ *Strix aluco* habitat selection models in Jadar and Radjevina microregion, Podrinje area, Western Serbia.

Model No	Model Variables	AIC	Δ*_i_*AIC	Akaike *w _i_*	AUC	CV (%)
I	Cultivated land	3926.127	2.336	0.119	0.595	0.602
II	Suburban	4083.266	159.475	<0.001	0.515	0.602
III	Altitude	4079.353	155.562	<0.001	0.545	0.602
**IV**	**Cultivated land + Suburban**	**3923.791**	**0**	**3.841**	**0.601**	**0.602**
V	Cultivated land + Altitude	3924.980	1.189	0.212	0.607	0.602
VI	Suburban + Altitude	4059.943	136.152	<0.001	0.566	0.602
VII	Cultivated land + Suburban + Altitude	3924.391	0.600	0.284	0.607	0.602

AIC is Akaike’s information criterion; Δ*_i_*AIC is (AIC)*_i_* − (AIC)_min_; Akaike *w _i_* is the Akaike weight; AUC is the area under the curve; CV is the percent of cases accurately predicted in cross validation. The best model is bolded.

**Table 5:** Coefficient estimates and statistical significance for top model (Model No IV) of tawny owl *Strix aluco* habitat selection analysis.

Covariate	Estimate	SE	z	*p* Value
(Intercept)	0.263	0.043	6.117	<0.001
Suburban	0.355	0.173	2.054	0.040
Cultivated land	−1.231	0.101	−12.118	<0.001

## Data Availability

The GPS fixes and other raw data supporting the results and conclusions of this article will be made available by the corresponding author upon request.
